# Evaluation of Chitosan-Microcrystalline Cellulose Blends as Direct Compression Excipients

**DOI:** 10.1155/2017/8563858

**Published:** 2017-12-19

**Authors:** Emmanuel O. Olorunsola, Grace A. Akpan, Michael U. Adikwu

**Affiliations:** ^1^Department of Pharmaceutics and Pharmaceutical Technology, University of Uyo, Uyo, Nigeria; ^2^University of Abuja, Abuja, Nigeria

## Abstract

This study was aimed at evaluating chitosan-microcrystalline cellulose blends as direct compression excipients. Crab shell chitosan, *α*-lactose monohydrate, and microcrystalline cellulose powders were characterized. Blends of the microcrystalline cellulose and chitosan in ratios 9 : 1, 4 : 1, 2 : 1, and 1 : 1 as direct compression excipients were made to constitute 60% of metronidazole tablets. Similar tablets containing blends of the microcrystalline cellulose and *α*-lactose monohydrate as well as those containing pure microcrystalline cellulose were also produced. The compact density, tensile strength, porosity, disintegration time, and dissolution rate of tablets were determined. Chitosan had higher moisture content (7.66%) and higher moisture sorption capacity (1.33%) compared to microcrystalline cellulose and lactose. It also showed better flow properties (Carr's index of 18.9% and Hausner's ratio of 1.23). Compact density of tablets increased but tensile strength decreased with increase in the proportion of chitosan in the binary mixtures. In contrast to lactose, the disintegration time increased and the dissolution rate decreased with increase in the proportion of chitosan. This study has shown that chitosan promotes flowability of powder mix and rapid disintegration of tablet. However, incorporation of equal proportions of microcrystalline cellulose and chitosan leads to production of extended-release tablet. Therefore, chitosan promotes tablet disintegration at low concentration and enables extended-release at higher concentration.

## 1. Introduction

Direct compression is the most preferred of all methods of tableting as it saves both time and energy [1]. In this method, tablets are compressed directly from the powder blends of active ingredients and suitable excipients [2]. Direct compression is suitable for moisture and heat-sensitive materials. However, it has some disadvantages such as possibility of particle size stratification which can lead to poor content uniformity, difficulty in formulation of large dose drugs, generation of static charge while handling the dry materials, and possible interaction between the diluents and the drug [3].

The desired properties of excipients for direct compression are powder fluidity, good compressibility, low moisture sensitivity, and rapid disintegration action [2, 4]. Combination of two or more excipients with different desirable properties enhances the manufacturing process and/or product performance. The quality of a delivery system is determined by its manufacturing process and the performance of the dosage form [5].

Microcrystalline cellulose (MCC) is a white crystalline powder composed of agglomerated porous microfibres [6]. The popularity of this excipient in direct compression is due to its extremely good bonding properties as a dry binder and formation of tablets with good mechanical strength. However, its flow properties are relatively poor because of its small particle size; and it exhibits low bulk density because of its particle shape [7]. Larger particles of this polymer have better flowability and lubricity but lower compressibility [7]. Since microcrystalline cellulose has some limitations, addition of another excipient that will augment the required properties for direct compression is desirable.

Chitosan (CTS) is used to solve the problem associated with dissolution rate and bioavailability of poorly water-soluble drugs [8]. The work of Sawayanagi et al. [9] suggested that incorporation of chitosan as excipient in direct compression could improve powder flow. Hence, the desirable properties of chitosan for direct compression are powder fluidity and rapid disintegration action. Previous studies, however, showed the limitation of chitosan as being production of tablets with low mechanical strength [10].

Microcrystalline cellulose and chitosan have different desired properties for direct compression though each one has its own limitation. Combination of both polymers at different proportions for use as direct compression excipients will produce tablets of different properties and quality. This paper reports the interplay of the two polymers combined at different proportions in optimizing direct compression process. Metronidazole was used as a model drug because on its own it has poor compressibility and compactability [1]. The work attempts to utilize the characters of chitosan to enhance the tablet manufacture and formulation performance of a poorly compressible drug produced by direct compression.

## 2. Materials and Methods

### 2.1. Materials

The materials used include metronidazole powder (Hopkins and Williams, England), Merck Art 2330 microcrystalline cellulose (E. merck, Darmstadt), and chitosan obtained from shells of* Callinectes gladiator* having degree of deacetylation of 62.7% and viscosity of 2.88 mPas at 2% concentration, spindle #1, and 60 rpm [11]. Others are *α*-lactose monohydrate (Riedel De Haen Seelze, Hannover), magnesium stearate (BDH Poole, England), and talc (BDH Poole, England).

### 2.2. Fourier Transform Infrared (FTIR) Spectroscopy

A sample each of metronidazole, chitosan, microcrystalline cellulose, and physical mixture of the three materials was prepared in a potassium bromide disk in a hydrostatic press at 6 to 8 tons pressure. The FTIR spectrum of each sample was recorded at scanning range of 350 to 4000 cm^−1^ using a spectrophotometer (Model 8400S, Shimadzu Corporation, Kyoto-Japan).

### 2.3. Physicochemical Characterization of the Direct Compression Excipients

#### 2.3.1. Moisture Content

One gramme sample (chitosan, microcrystalline cellulose, and lactose) was transferred into different Petri dish and then dried in an oven (Gallenkamp, Germany) at 105°C until a constant weight was obtained. The percentage moisture content was then determined as the ratio of weight loss to weight of sample expressed as percentage [12].

#### 2.3.2. Moisture Sorption Capacity

One gramme sample (chitosan, microcrystalline cellulose, and lactose) was separately weighed and distributed evenly over the surface of different Petri dish. The sample was then placed in a large desiccator containing distilled water in its reservoir at room temperature and relative humidity of 75%. The weight gained by the exposed sample over a 5-day period was recorded and the amount of water absorbed was calculated from the weight difference [12].

#### 2.3.3. Densities

A 10 g sample was placed in a 50 ml measuring cylinder and the bulk volume was taken. The system was tapped 50 times after which the volume was retaken. The bulk density (BD) and tapped density (TD) were calculated as the ratio of mass to the corresponding volume [13].

The true density (*D*_*t*_) of each of the three samples (chitosan, microcrystalline cellulose, and lactose) was determined using the specific gravity bottle method. A clean, dry 25 ml specific gravity bottle was filled with xylene and its weight was determined. Some of the xylene was poured out and 1 g sample was placed inside. More xylene was added until the bottle was filled and was wiped dry of excess fluid. Its weight was again determined. The true density (*D*_*t*_) was calculated using the following equation:

(1) $$\begin{array}{c} D_{t}=\frac{w}{a+w-b}\times SG, \end{array}$$

where *w* is the weight of powder, *a* is weight of bottle + xylene; *b* is weight of bottle + solvent + powder, and SG is specific gravity of xylene.

#### 2.3.4. Flow Properties

The compressibility index (Cl) and Hausner's ratio (HR) were calculated using the formulae:

(2) CI=TD−BDTD×100%

(3) HR=TDBD.

#### 2.3.5. Hydration Capacity

One gramme sample was placed in a test tube and 10 ml of distilled water added and then stoppered. The content was mixed on a vortex mixer for 2 min. The mixture was allowed to stand for 10 min and immediately centrifuged at 1000 rev/min for 10 min. The supernatant was carefully decanted and the sediment was weighed. The hydration capacity was calculated as the ratio of weight of water uptake to weight of dry sample.

#### 2.3.6. Swelling Capacity

One gramme sample was put into a 10 ml graduated cylinder and 10 ml of distilled water was poured into the cylinder and mixed over a vortex mixer for 2 min. The suspension was allowed to stand for 30 min and the new volume of the hydrated excipient was taken. The swelling capacity (SC) was computed according to the following equation:

(4) $$\begin{array}{c} SC=\frac{V_{2}-V_{1}}{V_{1}}\times 100\%, \end{array}$$

where *V*_2_ is the volume of the hydrated or swollen material and *V*_1_ is the tapped volume of the material prior to hydration.

### 2.4. Tablet Formulation

Nine batches of 100 tablets each were prepared based on Table 1. Each tablet was made to contain 200 mg metronidazole, 300 mg direct compression excipient, and 2.5 mg each of talc and magnesium stearate. Batch 1 was made to contain 100% microcrystalline cellulose as the direct compression excipient and batches 2 to 5 were made to contain microcrystalline cellulose and chitosan in ratios 9 : 1, 4 : 1, 2 : 1, and 1 : 1 respectively while batches 6 to 9 were made to contain microcrystalline cellulose and lactose in ratios 9 : 1, 4 : 1, 2 : 1, and 1 : 1, respectively, as the direct compression excipient.

Metronidazole and the direct compression excipients for each batch were weighed individually and triturated for 5 min. Talc and magnesium stearate were also weighed separately and blended with the powder mix for further 5 min. Tablets were produced by compression at 15 KN (which was predetermined as compression pressure to produce tablets of crushing strength in the neighbourhood of 4–7 kgf) using F_3_ single punch tableting machine (Cadmach Machinery Co. PVT., India) fitted with 12.5 mm punches.

### 2.5. Tablet Evaluation

#### 2.5.1. Compact Density

Five tablets were selected at random from each batch and weighed individually using an analytical balance (Mettler, Germany). The diameter and thickness of the tablets were also determined using a micrometer screw gauge. The compact density (CD) was determined using the following equation:

(5) $$\begin{array}{c} CD=\frac{m}{\pi r^{2}h}, \end{array}$$

where *m* is the mass of tablet, *r* is the radius, and *h* is the thickness.

#### 2.5.2. Tensile Strength

The crushing strength (*F*) of the five selected tablets per batch was determined using Monsanto hardness tester. The tensile strength (TS) was determined using the value of crushing strength and the corresponding values of diameter and thickness obtained in Section 2.5.1.

(6) $$\begin{array}{c} TS=\frac{2F}{\pi dt}, \end{array}$$

where *F* is the crushing strength, *d* is the diameter, and *t* is the thickness of tablets.

#### 2.5.3. Porosity

The tablet porosity Φ was determined using

(7) $$\begin{array}{c} \Phi =1-\frac{CD}{D_{t}}, \end{array}$$

where CD is compact (tablet) density and *D*_*t*_ is true density of the powder blend.

The true density of the powder blends was determined using specific gravity bottle method as described under Section 2.3.3. One gramme sample of each powder blend was used for this procedure. The true density was calculated using (1) which is already stated under the section.

#### 2.5.4. Disintegration Time

Six tablets were selected at random from each batch and subjected to disintegration test in distilled water maintained at 37°C using disintegration apparatus (Erweka, Germany). The disintegration time was taken to be the time when no particle remained in the basket of the disintegration apparatus.

#### 2.5.5. Dissolution Rate

*In vitro* dissolution test for three tablets each from batches F1, F2, F5, F6, and F9 was carried out using the USP dissolution apparatus. A tablet was placed in a wire mesh basket suspended in a dissolution medium of 900 ml of 0.1 N HCl constantly maintained at 37 ± 1°C. The basket was rotated at a speed of 50 rpm and the experiment was allowed for 3 h. A 10 ml aliquot was withdrawn at 10 min intervals for the first 1 h and then at 30 min intervals for the following 2 h. Each sample was filtered through Whatman filter paper number 1 and the absorbance was taken at 278 nm using UV spectrophotometer (Jenway, England). A graph of cumulative % drug released was plotted against time.

### 2.6. Statistical Analysis

Data were expressed as mean ± standard error of mean. Statistical analysis was done using one-way analysis of variance followed by Turkey-Kramer multiple comparison test using GraphPad Instat-3 software. Significance of difference was set at* p values* less than 0.05.

## 3. Results

### 3.1. Fourier Transform Infrared Spectra

**Figure 1:**
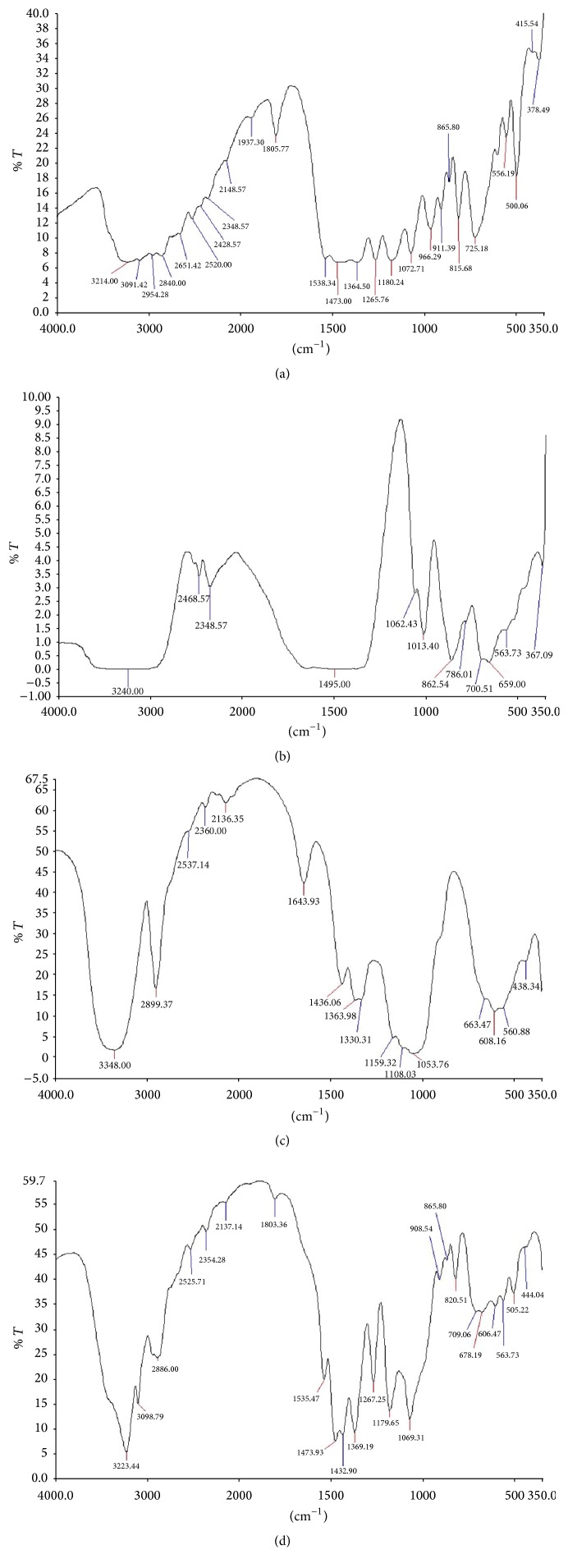
FTIR spectra of (a) metronidazole, (b) chitosan, (c) microcrystalline cellulose, and (d) physical mixture.

The spectra of the pure ingredients and the physical mixture are shown in Figures 1(a)–1(d). The spectrum of metronidazole (Figure 1(a)) shows unique peaks at 1556.34, 1475.00, 1364.30, 1072.71, 911.59, and 865.50 cm^−1^. The FTIR spectrum of chitosan (Figure 1(b)) showed 10 major peaks and 2 bands lying between 367.09 and 3240.00 cm^−1^ while that of microcrystalline cellulose (Figure 1(c)) showed 16 peaks lying between 438.34 and 3348.00 cm^−1^. All the major peaks found in the spectrum of the physical mixture were obtainable in at least one of the individual components. The only differences were in the location and intensity of the peaks.

### 3.2. Physicochemical Properties of the Direct Compression Excipients

Some physicochemical properties of chitosan, lactose, and microcrystalline cellulose are shown in Table 2. Chitosan was observed to have the highest moisture content and the highest moisture sorption capacity. It also had the highest bulk and true densities, the values being significantly higher than those of the other two polymers. Hausner's ratio and Carr's index followed the same trend as chitosan < microcrystalline cellulose < lactose. The hydration and swelling capacities followed the same trend of microcrystalline cellulose > chitosan > lactose.

### 3.3. Physicomechanical Properties of the Tablets

The physical and mechanical properties of the tablets are shown in Table 3. The compact density increased with increase in the proportion of chitosan (F2 to F5) but decreased with increase in the proportion of lactose (F6 to F9). There was a significant difference in the tensile strength of tablets (*p* < 0.001). Formulation F1 had the highest tensile strength. The tensile strength decreased with increase in the proportion of chitosan in the binary mixture (F2 to F5). It also decreased with increase in the proportion of lactose in the binary mixture of the direct compression excipient. The decrease in tensile strength was more with chitosan.

Porosity values initially increased and afterwards decreased with increase in the proportion chitosan and decreased with increase in the proportion of lactose. However, the differences in the porosity values were not significantly different. Formulation F2 had the shortest disintegration time. The disintegration time increased with increase in the proportion of chitosan in the direct compression excipient (*p* < 0.0001) but decreased with increase in the proportion of lactose. There was incomplete tablet disintegration of F5 at 60 min.

### 3.4. Dissolution Profile

**Figure 2:**
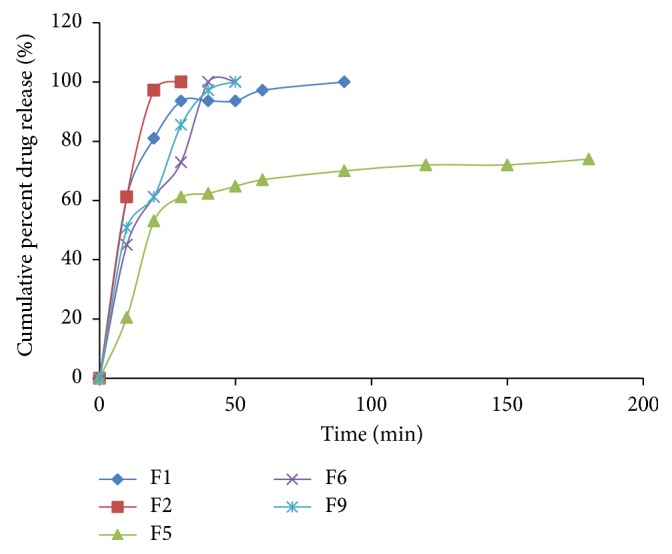
Plot of cumulative percent drug release versus time.

The plot of cumulative percent drug released versus time for batches F1, F2, F5, F6, and F9 is shown in Figure 2. Formulation F2 brought forth the fastest drug release with 97.2% drug release within 20 min. Formulation F5 had very slow dissolution rate and only released 74% of the labeled drug at 180 min. For formulations containing lactose, dissolution rate increased with increase in the proportion of lactose.

## 4. Discussion

The absence of new peak in the spectrum of the physical mixture shows that the ingredients are compatible. Moisture plays an important role in the consolidation and compaction of pharmaceutical powders; and the changes that occur in these processes are as a result of the effect of moisture on the interparticle and intermolecular forces [14]. Interaction between water and polymer also occurs leading to a change in the behaviour which alters the moisture action from that of a good tablet binder to a factor responsible for the weak tensile strength of tablets [15]. The moisture content of the crab shell chitosan is within the 10% which is permissible for chitosan [16]. Since presence of water has negative effects on compressibility of powders and tensile strength of tablets, incorporation of microcrystalline cellulose (having lower moisture content) is necessary to ensure adequate and effective compression of the powder mix.

The bulk density of powder is an indicator of powder's ability to undergo compression. Low bulk density implies high tendency of densification. It is attributable to high particulate irregularities and a highly porous structure [17]. From the characterization of the direct compression excipients, microcrystalline cellulose had the lowest bulk density showing its highest tendency of undergoing compression. Chitosan had the highest true density. The true density of a powder is a property that excludes every void space and it shows the extent to which a powder can be compressed. Therefore, a blend of microcrystalline cellulose and chitosan is superior to the individual constituent in terms of compressibility.

Carr's compressibility index and Hausner's ratio are used for predicting powder flow characteristics. They are indirect measures of bulk density, size and shape, moisture content, and cohesiveness of materials as all these parameters can influence compressibility. Hausner's ratio of less than 1.25 indicates good flow and 1.25–1.50 indicates passable flow while greater than 1.50 indicates poor flow [18]. Based on Hausner's ratio, the flow of chitosan is good and microcrystalline cellulose is passable while lactose is poor. Also, Carr's index of less than 18% indicates good flow and 18–33% indicates passable flow while greater than 33% indicates poor flow [18]. Based on this index, chitosan and microcrystalline cellulose have passable flow while lactose has a poor flow. Therefore, flow of powder mix is better enhanced when MCC is combined with chitosan compared to when it is combined with lactose. Powder must flow easily and uniformly into the die cavity to ensure tablet weight uniformity and production of tablet with consistent and reproducible properties.

An ideal disintegrant should have poor solubility, poor gel formation, and good hydration capacity [19]. The hydration capacity of chitosan cannot be compared to lactose since the latter is very water soluble (Table 2). However, chitosan can be said to be superior to lactose as a codiluent in direct compression since rapid disintegration action which is also related to poor solubility is one of the ideal requirements of a direct compression excipient [2]. Moisture sorption and water uptake had been found to be the major mechanisms of disintegration for chitosan [19].

Swelling is another indication of tablet disintegration ability. The result showed that swelling capacity of microcrystalline cellulose is higher than that of chitosan. However, it is difficult to make an assertion or a comparison in respect of the disintegrant action of the two polymers based on their swelling capacities. This is because while microcrystalline cellulose causes rapid tablet disintegration by swelling, chitosan causes rapid tablet disintegration by capillary action [19]. Lactose as a codiluent in direct compression has poor disintegrant action because of its extremely poor hydration and swelling.

On application of pressure to powder bed, the bulk volume of the powder is reduced leading to increase in bulk density [20]. On reaching a sufficient pressure, a compact (tablet) is formed. In this study, the compact density of tablet increased with increase in the proportion of chitosan (F2 to F5) but decreased with increase in the proportion of lactose (F6 to F9). These phenomena are, respectively, explained by the plastic deformation behaviour of chitosan [21] and the fragmentation behaviour of lactose [22] upon compaction. The difference in the compact density is also directly related to the true density of the codiluent. The preliminary characterization of the excipients showed the true density of chitosan to be the highest. Formulation F5 had the highest compact density (smallest volume) because it contains the highest amount of chitosan. A small-sized tablet facilitates swallowing.

The mechanical strength of tablets produced upon compression is the most essential requirement of direct compression excipients [1]. Formulation F1 (which contained 100% MCC as direct compression excipient) had the highest tensile strength. Incorporation of both chitosan and lactose as codiluents caused decrease in tensile strength. Chitosan is inferior to lactose as a codiluent in this regard as it caused greater reduction in tablet strength.

Tablet porosity is influenced by the volume of reduction as compaction pressure is applied [20]. Tablet properties such as mechanical strength and disintegration time are in turn affected by pore structure of powder. Chitosan had the highest true density (Table 2) and it deforms plastically upon compaction [21]. These factors explain why porosity reduced as the proportion of the polymer was increased for batches F2 to F5. The insignificant difference in the true density of lactose and microcrystalline cellulose (Table 2) as well as fragmentation of lactose rather than plastic deformation upon compaction [22] explains the insignificant difference in porosity of tablets (F6 to F9) containing different proportions of the two excipients.

Tablets should have sufficient strength to withstand the mechanical shocks during the tablets manufacturing, packaging, and transportation, yet they should be soft enough to be able to disintegrate properly after being swallowed. Tablets of low porosity tend to have high mechanical strength and long disintegration time [23]. Of the formulations F2 to F5, F2 had the highest porosity, yet it had the highest tensile strength and the shortest disintegration time. This does not conform to the report of Juppo [23]. The short disintegration time is justified by the high porosity but the high tensile strength can only be justified by the high proportion of MCC with the characteristic high bonding strength. Formulation F2 (which contains MCC and CTS in ratio 9 : 1) is a fast disintegrating tablet.

The disintegration time increased with increase in the proportion of chitosan in the direct compression excipients (*p* < 0.0001) but decreased with increase in the proportion of lactose. This is an anomalous behaviour since chitosan already behaved as a superdisintegrant at low concentration. Formulations F3 and F4 passed the disintegration test for normal (immediate-release) tablets but F5 (which contains MCC and CTS in ratio 1 : 1) failed the test having a disintegration time above 60 min. This is in agreement with the fact that chitosan is useful for controlled-release formulation [24].

Formulations F1, F2, F6, and F9 satisfied the United States Pharmacopoeia requirement of 75% drug release within 1 h [25]. Therefore, the excipient combinations at those proportions are suitable for production of immediate-release tablets. Formulation F5 (which contains the highest proportion of chitosan) brought forth slow drug release of 74% of the labeled drug in 180 min. The high concentration of chitosan in this formulation is responsible for the extended-release of the drug [24]. The metronidazole tablets showed different in vitro drug release, which may affect bioavailability. The results indicated that the concentration of chitosan in the tablet formulations has a significant effect on the mechanical and release properties of the tablets.

## 5. Conclusion

Chitosan is characterized by good flowability and rapid disintegration action, properties that are desired of direct compression excipients. It is, however, limited by poor mechanical properties. It is superior to lactose as a codiluent in direct compression. This work has also shown that chitosan has a good disintegrant property when used at low concentration and significant controlled drug release when used at high concentration. It has a significant impact on tablet disintegration and drug dissolution. Chitosan, with such properties, has many applications in drug delivery. In combination with microcrystalline cellulose as direct compression excipient, it can be used at low concentration for manufacture of fast disintegrating tablet, at moderate concentration for immediate-release tablet, and at high concentration for controlled-release tablet.

## Figures and Tables

**Table 1:** Tablet formula.

Ingredient/tablet	F1	F2	F3	F4	F5	F6	F7	F8	F9
Metronidazole (mg)	200.0	200.0	200.0	200.0	200.0	200.0	200.0	200.0	200.0
MCC (mg)	300.0	270.0	240.0	200.0	150.0	270.0	240.0	200.0	150.0
Chitosan (mg)	0.0	30.0	60.0	100.0	150.0	0.0	0.0	0.0	0.0
Lactose (mg)	0.0	0.0	0.0	0.0	0.0	30.0	60.0	100.0	150.0
Mg stearate (mg)	2.5	2.5	2.5	2.5	2.5	2.5	2.5	2.5	2.5
Talc (mg)	2.5	2.5	2.5	2.5	2.5	2.5	2.5	2.5	2.5

Tablet weight (mg)	505.0	505.0	505.0	505.0	505.0	505.0	505.0	505.0	505.0

MCC: microcrystalline cellulose.

**Table 2:** Some physicochemical properties of chitosan, lactose, and microcrystalline cellulose.

Parameters	Chitosan	Lactose	Microcrystalline cellulose
Moisture content (%)	7.66 ± 0.98	2.00 ± 0.47	1.00 ± 0.00
Moisture sorption capacity (%)	1.33 ± 0.05	0.36 ± 0.04	0.21 ± 0.01
Bulk density (g/cm^3^)	0.60 ± 0.01	0.51 ± 0.01	0.32 ± 0.00
Tapped density (g/cm^3^)	0.74 ± 0.01	0.77 ± 0.00	0.44 ± 0.01
True density (g/cm^3^)	1.74	1.45	1.45
Carr's index (%)	18.90	33.90	28.20
Hausner's ratio	1.23	1.51	1.39
Hydration capacity	0.60 ± 0.01	0.05 ± 0.01	2.23 ± 0.02
Swelling capacity (%)	14.29 ± 0.00	Dissolved completely	39.78 ± 1.55

**Table 3:** Physical and mechanical properties of tablets.

Batches	Compact density (g/cm^3^)	Tensile strength (kg/cm^2^)	Tablet porosity	Disintegration time (min)
F1	1.18 ± 0.01	8.65 ± 0.71	0.45 ± 0.02	2.43 ± 0.07
F2	1.01 ± 0.02	4.33 ± 0.41	0.49 ± 0.01	0.38 ± 0.05
F3	1.04 ± 0.02	4.33 ± 0.41	0.48 ± 0.01	1.08 ± 0.23
F4	1.20 ± 0.02	4.15 ± 0.52	0.45 ± 0.01	2.12 ± 0.48
F5	1.23 ± 0.02	3.49 ± 0.26	0.40 ± 0.01	>60
F6	1.16 ± 0.03	7.54 ± 0.77	0.43 ± 0.01	2.38 ± 0.04
F7	1.15 ± 0.01	6.93 ± 0.75	0.41 ± 0.02	2.09 ± 0.30
F8	1.12 ± 0.03	6.64 ± 0.95	0.41 ± 0.02	1.18 ± 0.30
F9	1.09 ± 0.03	6.10 ± 0.32	0.40 ± 0.00	0.42 ± 0.04
